# Dance-communicated distances support nectar foraging as a supply-driven system

**DOI:** 10.1098/rsbl.2022.0155

**Published:** 2022-08-31

**Authors:** Bradley D. Ohlinger, Roger Schürch, Mary R. Silliman, Taylor N. Steele, Margaret J. Couvillon

**Affiliations:** Department of Entomology, Virginia Polytechnic Institute and State University, Blacksburg, VA, USA

**Keywords:** honeybee foraging, waggle dance, supply-driven, demand-driven

## Abstract

Much like human consumers, honeybees adjust their behaviours based on resources' supply and demand. For both, interactions occur in fluctuating conditions. Honeybees weigh the cost of flight against the benefit of nectar and pollen, which are nutritionally distinct resources that serve different purposes: bees collect nectar continuously to build large honey stores for overwintering, but they collect pollen intermittently to build modest stores for brood production periods. Therefore, nectar foraging can be considered a supply-driven process, whereas pollen foraging is demand-driven. Here we compared the foraging distances, communicated by waggle dances and serving as a proxy for cost, for nectar and pollen in three ecologically distinct landscapes in Virginia. We found that honeybees foraged for nectar at distances 14% further than for pollen across all three sites (*n* = 6224 dances, *p* < 0.001). Specific temporal dynamics reveal that monthly nectar foraging occurs at greater distances compared with pollen foraging 85% of the time. Our results strongly suggest that honeybee foraging cost dynamics are consistent with nectar supply-driven and pollen demand-driven processes.

## 1. Introduction

In commerce, supply and demand interact to determine the market value of goods and services. Consequently, supply chains are managed to produce at rates and prices that profitably meet consumer demand [1]. Supply/demand ratios modulate the consumer cost dynamics and influence consumer decisions [1]. Meanwhile, these processes operate in a fluctuating market. Analogously, the foraging landscape navigated by animals also fluctuates, with dynamic shifts in both supply and demand then modulating food-collection behaviours. Unsurprisingly, many of the terms used in consumer economics have been co-opted to animal foraging ecology [2,3].

Honeybees are highly efficient social foragers that can survey complex landscapes, identify attractive resources and allocate their foraging efforts according to food quality and colony needs by selectively recruiting to the best resources at any given time [4–9]. Recruitment is accomplished via the waggle dance, where a successfully returning forager who has found a good source of food performs a stereotyped behaviour that encodes the distance and direction from the hive to the forage [3,10]. Workers that follow a dance can then use the information to find the advertised food [10,11]. Lastly, honeybee foragers, like human consumers, respond to supply and demand forces as they collect resources [3].

Honeybees' most important food resources are pollen, a source of protein and lipids that is fed to developing brood, and nectar, a source of carbohydrates that is turned into honey, which is mostly food for adult bees. Honeybees in temperate regions must also, during the foraging season (spring–autumn), create large stockpiles of honey that serve as food for the winter bees that engage in energetically costly thermoregulation [12] and are critical to colony overwintering survival [13,15]. Honeybees therefore are strongly motivated to collect nectar and will continuously do so even if the colony already possesses honey stores [16]. Nectar foraging, therefore, is considered supply-driven because the amount coming into the colony is only limited by its availability in the environment [3]. By contrast, pollen foraging is considered demand-driven, where the amount of pollen coming into the hive is also strongly modulated by colony needs because pollen is required when brood is actively being reared [8,9,17,18].

Decoding protocols to analyse honeybee waggle dances recover the distance and direction to the forage as discrete components [19–21]. This is useful, as the encoded distance information can act as a proxy for forage availability [22]. Honeybees are economic foragers [7], and flight is costly [23,24], so foragers will only recruit nest-mates to resources as far as necessary [22,25,26]. In other words, increases in communicated foraging distance indicate decreases in forage [22,25,26].

Although terms like supply and demand have long been applied to bee foraging ecology [3], the cost dynamics in supply versus demand-driven systems remain less explored. Flight distance, as a large cost associated with resource collection, is analogous to consumer prices [7,22,24] and both should respond similarly to fluctuating supply/demand ratios. Supply-driven markets, used by foragers/consumers with continuous resource demand, and demand-driven markets, used by foragers/consumers with intermittent resource demand, should produce distinct consumer/foraging responses. Foragers/consumers are expected to respond more strongly and more consistently to resource availability changes in supply-driven processes than in demand-driven processes. Therefore, one would predict that communicated honeybee foraging distances should be inversely proportional to nectar availability, as nectar collection is considered supply-driven (i.e. honeybees always need nectar). By contrast, honeybee foraging distances should only be inversely proportional to pollen availability when pollen demand is high. Additionally, the pollen dancers' communicated distances should be lower than nectar distances.

Here we investigate whether foraging distances, as communicated by the waggle dances, support supply-driven nectar foraging and demand-driven pollen foraging. We analysed 6224 waggle dance distances, which reflect cost and are an availability proxy, from bees in three ecologically distinct landscapes in Virginia to determine overall and monthly communicated foraging distance for both nectar and pollen.

## 2. Material and methods

We studied nine predominately *Apis mellifera lingustica* colonies, each consisting of a queen and approximately 5000 workers, at three sites across Virginia, with three hives per site. We housed colonies in glass-walled observation hives composed of three American Standard Deep Langstroth frames. The glass provided an unimpeded view of behaviours, including dances. We maintained the hives indoors at the Prices Fork Research Center (PFRC; 37.21148, −80.48935) in Blacksburg, Virginia, the Tidewater Agricultural Research and Extension Center (TAREC; 36.66447, −76.73278) in Suffolk, Virginia and the Alson H. Smith Jr. Agricultural Research Center (WAREC; 39.11349, −78.28449) in Winchester, Virginia. Foragers were able to enter/exit colonies through a 5 cm × 30 cm PVC piping from the colony entrance to the outside. We provided the colonies with supplemental sucrose solution during times of forage dearth and to maintain consistent food stores. The landscapes surrounding the three sites provided unique ecological contexts: TAREC consisted of row croplands, WAREC of orchard croplands and PFRC of a mix of residential, agricultural and semi-natural lands.

We video recorded and decoded waggle dances using an updated protocol developed by Couvillon *et al*. [19]. Briefly, we decoded four waggle runs (information-rich, repeated subunits) per dance to extract run duration, which encodes the distance [10]. We used frame-by-frame playback for videos recorded on 177 days from 13 April to 31 October 2018 and from 10 April to 18 October 2019. We noted whether the dancer was carrying pollen, which is highly visible in the videos. Although presumably some non-pollen dancers might be recruiting for water, this usually represents less than 5% of the overall foraging effort [3,25]. In all, we decoded 6224 dances, with 1931 (nectar: 1144, pollen: 787) at PFRC, 2282 (nectar: 1329, pollen: 953) at TAREC and 2011 at WAREC (nectar: 1273, pollen: 738).

We used the methods reported in Schürch *et al*. [21] to convert durations into distances by using bootstrap sampling from the universal calibration dataset, consisting of run durations to known distances [21] and has been shown to perform well across landscapes and contexts [27]. The method also reflects the uncertainty inherent in the communication [19–21,28]. To identify temporal trends in communicated foraging distances, for each dance we simulated the distances 1000 times and then calculated the median simulated distance. Then we determined the effect of month and forage type on distance at three sites by using log-transformed linear mixed models from the Lme4 package [29], with distance as a response variable; month, site, forage type and the first- and second-order interactions as fixed effects; and hive as a random effect. We used R 4.1.1 for all analyses [30] and we obtained the estimated marginal means (EMM) using the emmeans package [31].

## 3. Results

**Figure 1.**
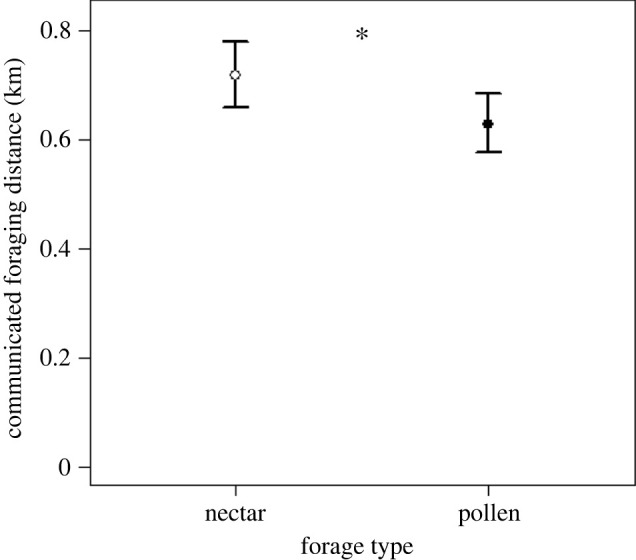
Nectar foraging distances, as communicated by waggle dances, were significantly (*) greater than pollen foraging distances across all three sites (*n* = 6224 dances). White circles are the EMM for nectar and black circles are the EMM for pollen, with the bars representing the 95% confidence intervals.

Across all the dances (*n* = 6224), we found a significant effect of the interactions among month, forage type and site (LRT = 81.05, d.f. = 12, *p* < 0.001). We observed a significant effect of forage type on communicated foraging distance, with nectar foragers recruiting significantly further away, 13.9%, relative to pollen (nectar: EMM = 717.2 m, 95% CI [659.4 m, 779.9 m]; pollen: EMM = 629.3 m, 95% CI [577.2 m, 685.9 m]; mean difference = 87.9 m, 95% CI [58.4 m, 116.1 m], *p* < 0.001; figure 1). There were some site-specific differences, with communicated nectar distances reflecting the overall result at PFRC (mean difference = 125.7 m, 95% CI [64.6 m, 181.5 m], *p* < 0.001) and TAREC (mean difference = 115.8 m, 95% CI (75.3 m, 153.5 m], *p* < 0.001). At WAREC, the communicated nectar distance was higher, but not significantly so (mean difference = 21.5 m, 95% CI [−33.3 m, 72.2 m], *p* = 0.422).

**Figure 2.**
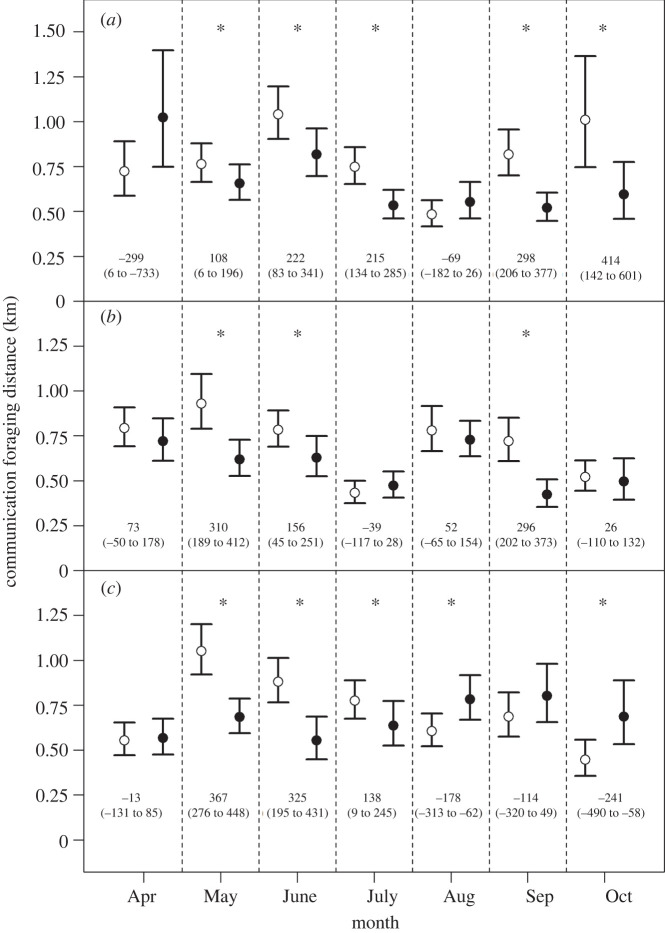
Month and forage type and their interactions affect communicated distance at the three sites: PFRC (*a*), TAREC (*b*) and WAREC (*c*). Significant differences between communicated foraging distance by resource type (nectar = white, pollen = black) is indicated by asterisks (*). White circles are the EMM for nectar and black circles are the EMM for pollen, with the bars representing the 95% confidence intervals. When a significant difference (*) existed between resources' foraging distance, nectar was greater at PFRC, TAREC, and for three of the five months at WAREC. Mean differences (metres) with 95% CI are reported in the margins.

In our monthly/site specific investigations, when there were significant differences between monthly communicated foraging distance for nectar versus pollen, communicated nectar distances were higher in 11 of the 13 months, or 84.6% (figure 2). Specifically, nectar was always higher at PFRC (figure 2*a*) and TAREC (figure 2*b*). At WAREC, overall nectar distances were significantly higher than pollen, as was seen in May, June and July; pollen distances were higher in August and October (figure 2*c*).

## 4. Discussion

Here we investigated honeybee foraging distance, as communicated by waggle dances, for nectar versus pollen across two foraging seasons in three distinct landscapes. We report that nectar versus pollen foraging distances were higher with our overall, site-specific, and monthly mean analysis. Our results suggest that the cost dynamics of nectar foragers are consistent with a supply-driven scenario, while that of pollen foragers are consistent with a demand-driven scenario.

In their decision to make a waggle dance, foragers weigh the energetic costs of flight against the energetic/nutritional content of food to efficiently meet their colony's nectar and pollen demands in dynamic environments [7]. Therefore, honeybees adjust their foraging efforts according to the supply of resources in their environment [22] and the demand for resources by their colony [3,8,9,17]. The supply of both nectar and pollen varies according to biotic and abiotic factors, such as season [22,32], competition [33], weather [34,35] and time of day [36].

However, the demand for nectar and pollen differs: honeybees keep modest stores of pollen [3,37] and increase pollen collection intermittently during periods of high brood production [8,9,17,38], while honeybees collect nectar continuously to meet their metabolic needs and to build large honey stores to buffer against nectar gaps and provide overwintering food [3,16]. We demonstrate that nectar foragers, compared to pollen foragers, displayed overall higher communicated distances (figure 1), a result that is likely driven by comparatively low foraging distances for pollen during periods with low pollen demand (i.e. when brood is not being reared). In other words, the colony does not need pollen during times of low demand and, consequently, is less willing to pay the ‘cost' of a further flight. Lastly, the overall result of higher nectar foraging distances is further supported by our site-specific analyses, which revealed significantly higher nectar distances at PFRC and TAREC and non-significantly higher nectar distances at the WAREC.

Why might WAREC be different? Incidentally, we observed a high number of colony and queen deaths at WAREC in 2019 (*n* = 7), even compared to 2018 (*n* = 2). Although the colonies were replaced as soon as possible, there was a small, unavoidable gap. Therefore, the non-significance at WAREC might be due to the high pollen demand in replacement colonies, as they experienced a break and then surge in brood rearing as new queens and/or colonies are introduced. Importantly, nectar distances were in fact significantly higher in 2018 (mean difference: 117.9 m, 95% CI [44.1 m, 183.2 m], *p* = 0.002), but not in 2019 (mean difference: −28.4 m, 95% CI [−125.7 m, 57.9 m], *p* = 0.527).

The temporal dynamics in foraging distance provide additional support for nectar as a supply-driven process: we observed significant differences in nectar and pollen distances in 13 of the 21 site/month combinations, with nectar foraging distances higher in 11 out of the 13 instances (figure 2). This effect is consistent with Couvillon *et al*. [25] and Balfour & Ratnieks [39], who reported that nectar dancers communicated longer foraging distances across 2 years in a rural and orchard system in England, respectively. Interestingly, some previous studies report either no difference in foraging distance for pollen and nectar [40] or longer pollen distances [41]. However, these studies used calibration models that relate waggle dance circuit duration (waggle run + return phase) to foraging distances and were completed before recent advancements demonstrating that run duration alone encodes distance, while the return phase duration responds to reward quality [42]. Therefore, these studies could be confounded by potential differences between the return phases of nectar and pollen foragers, but this possibility remains uninvestigated.

Pooled communicated foraging distances, where resource type is not distinguished, are commonly used as a proxy for resource availability [22,25,26,43]. Our study therefore fills a need to compare resource-specific trends in communicated distances. Such comparisons are particularly important given the distinct foraging economics of nectar and pollen foragers. The demand-driven economics of pollen foraging suggest that foragers might decrease foraging/recruitment effort when there is low pollen demand, and semi-field experiments show that pollen foragers will switch from low-quality pollen sources to high-quality nectar sources [44]. By contrast, nectar-specific trends in communicated foraging, which are likely supply-driven [3], might provide a better indicator of general forage availability.

Although we did not directly test that nectar foraging is a supply-driven process, our results are nonetheless consistent with cost dynamic predictions. Overall, these results suggest that forage-specific waggle dance data can more precisely assess the availability of pollen and nectar in landscapes than aggregated data.

## Data Availability

The data will be curated as a static dataset for use by other researchers and the public and will be stored at the Virginia Tech Data Repository (website: https://data.lib.vt.edu/, doi:10.7294/20498757).
